# HOXB7 acts as an oncogenic biomarker in head and neck squamous cell carcinoma

**DOI:** 10.1186/s12935-021-02093-6

**Published:** 2021-07-24

**Authors:** Xiang Wu, Jin Li, Tingyuan Yan, Xueping Ke, Xin Li, Yumin Zhu, Jianrong Yang, Zhongwu Li

**Affiliations:** 1 Department of Oral and Maxillofacial Surgery, The Affiliated Stomatological Hospital of Nanjing Medical University, Nanjing, 210029 Jiangsu China; 2 Jiangsu Province Key Laboratory of Oral Diseases, Nanjing Medical University, Nanjing, 210029 Jiangsu China

**Keywords:** Head and neck squamous cell carcinoma, HOXB7, Prognostic markers, Oncogene

## Abstract

**Background:**

The homeobox gene Homeobox B7 (HOXB7) is overexpressed across a range of cancers and promotes tumorigenesis through varying effects on proliferation, survival, migration and invasion. However, its expression pattern and oncogenic role of HOXB7 in head and neck squamous cell carcinoma (HNSCC) remain largely unexplored. Here, we aimed to explore the expression pattern of HOXB7, its clinical significance as well as functional roles in HNSCC.

**Methods:**

HOXB7 mRNA expression in HNSCC was determined by data mining and analyses from TCGA (The Cancer Genome Atlas) and GEO (Gene Expression Omnibus) datasets. The protein abundance of HOXB7 was measured by immunohistochemistry in 119 primary HNSCC samples and associations between its expression and clinicopathological parameters and patient survival were evaluated. The pro-tumorigenic roles of HOXB7 in HNSCC were further delineated in vitro by loss-of-function assay. And a xenograft tumor model was established in nude mice to assess the role of HOXB7 in tumor growth. Connectivity Map (CMap) analysis was performed to identify bioactive small molecules which might be potential inhibitors for HOXB7.

**Results:**

Bioinformatics analyses showed that HOXB7 mRNA was significantly overexpressed in 8 independent HNSCC datasets from TCGA and GEO databases. HOXB7 protein was markedly upregulated in HNSCC samples as compared to normal counterparts and its overexpression significantly associated with high pathological grade, advanced clinical stage, cervical node metastasis (*P* = 0.0195, 0.0152, 0.0300) and reduced overall and disease-free survival (*P* = 0.0014, 0.0007). Univariate and multivariate Cox regression analyses further revealed HOXB7 as an independent prognostic factor for patients’ overall survival. Moreover, HOXB7 knockdown significantly inhibited cell proliferation, migration and invasion and induced cell apoptosis in HNSCC cells, and resulted in compromised tumour growth in vivo. Furthermore, CMap (Connectivity map) analysis has identified three potential bioactive small molecule inhibitors (NU-1025, thiamine, vinburnine) for HOXB7 targeted therapy in HNSCC.

**Conclusions:**

Our findings revealed that overexpression of HOXB7 was associates with tumour aggressiveness and unfavourable prognosis by serving a novel prognostic biomarker in HNSCC. Moreover, HOXB7 might be involved in the development and progression of HNSCC as an oncogene, and thereby might be a potential therapeutic target for HNSCC.

**Supplementary Information:**

The online version contains supplementary material available at 10.1186/s12935-021-02093-6.

## Introduction

Head and neck squamous cell carcinoma (HNSCC) is a type of solid malignancy initiated from squamous epithelium and originated from oral cavity, larynx, and pharynx, the incidence of which ranks sixth in malignancy worldwide [1]. Smoking, drinking, chewing areca nut, and human papillomavirus infection are considered to be the most critical risk factors for HNSCC. Currently, HNSCC is treated with a combination of surgical resection, radiotherapy and chemotherapy, but the 5-year survival rate is not significantly improved [2, 3]. However, the validity and specificity of the traditional predictive parameters such as clinical stage, depth of invasion, surgical margin and involvement of cervical lymph nodes are relatively low and cannot meet the clinical need [2, 4, 5]. Therefore, it is important to find more accurate and reliable biomarkers for the early diagnosis, prognosis prediction and guidance for therapy selection for patients who were suffering from HNSCC, while, also contribute to the development of molecular targeted therapies for this deadly disease.

Burgeoning development of molecular biology research and genome-wide sequencing technology provide rich resources for biomarker development to better early diagnosis, patient stratification, personalized treatment as well as prognostic prediction [6, 7]. Homeobox (HOX) gene family are indispensable transcription factors in mammal embryonic development that regulate cell differentiation and morphogenesis. Every family member protein contains a consensus sequence—homeodomain, which encoded by a 61-amino-acids signature [8, 9]. HOX transcription factors are not only crucial for developmental process, but also play important roles in tumor initiation and progression [10]. Previous studies have reported that HOX genes promote cell proliferation, migration and invasion, epithelial–mesenchymal transition in several solid tumors including breast cancer, endometrial carcinoma and colorectal cancer [11–13].

It has been well documented that Homeobox B7 (HOXB7), a member of class I homeobox, plays an important role in tumorigenesis. Firstly, HOXB7 has been reported to be aberrantly expressed in various malignancies, such as oral cancer [14], lung cancer [15], breast cancer [16], gastric cancer [17], liver cancer [18] and esophageal cancer [19]. While, excessive overexpression of HOXB7 has highly confidential correlated with disease advancement and poor prognosis of esophageal squamous cell carcinoma and colorectal cancer [20, 21]. Secondly, several studies supported that HOXB7 might play a role in promotion of multistep process of tumor formation and progression, including proliferation, invasion, migration, angiogenesis and the epithelial–mesenchymal transition (EMT) [22–25]. On the other hand, some researchers observed a promoting role of HOXB7 in differentiation in hematopoietic stem cells and multipotent mesenchymal cells [26, 27]. These abovementioned data strongly suggest that HOXB7 might be a putative oncogene driving tumorigenesis and serves as a novel biomarker and promising therapeutic target. However, the expression pattern, clinical significance, and biological functions of HOXB7 in HNSCC remain largely unexplored yet.

In this study, we firstly confirmed that the expression of HOXB7 was significantly increased in HNSCC samples in publically available. Moreover, we determine the expression pattern of HOXB7 from a retrospective cohort of patients with primary HNSCC and reveal its clinicopathological and prognostic significance by immunohistochemistry (IHC). Secondly, we determined the tumorigenic roles of HOXB7 by loss-of-function assay and xenograft animal model in vitro and vivo assays. Finally, the GO (Gene Ontology) and KEGG (Kyoto Encyclopedia of Genes and Genomes) analysis were used to explore the biological functions of HOXB7-related genes. In addition, CMap (Connectivity map) analysis has identified three potential bioactive small molecule inhibitors for HOXB7 targeted therapy in HNSCC. Overall, our results revealed critical involvement of HOXB7 in HNSCC tumorigenesis and suggested that HOXB7 could be used as a novel prognostic biomarker and a potential therapeutic target for HNSCC.

## Materials and methods

### HNSCC samples

A total number of 119 tissue samples of patients with primary HNSCC were collected from patients who underwent curative resection (January 2012–September 2015) at the department of oral and maxillofacial surgery, Affiliated Stomatological Hospital of Nanjing Medical University, None of the patients had received adjuvant chemotherapy, radiation or any other treatment before resection, and all patients have detailed demographic, clinical, pathological and follow-up data. 26 normal adjacent oral mucosa samples obtained from donors during non-tumor surgeries were also included. This study was performed in accordance with guidelines outlined in the 1964 declaration of Helsinki and was approved by the Ethic Committee of Nanjing Medical University.

### Cell culture

Five HNSCC cell lines including SCC9, SCC25, Cal27, Fadu and a normal human oral keratinocytes (HOK) were obtained from American Type Culture Collection (ATCC, USA). And HN4 and HN6 were generously gifted from Dr. Wantao Chen (Shanghai Jiao Tong University). HNSCC cells were cultured in DMEM/F12 (Invitrogen, USA) with 10 % fetal bovine serum (FBS, Gbico, USA) in a humidified atmosphere with 5% CO_2_ at 37 °C.

### Small interfere RNAs, lentivirus production and cell transfection

The siRNAs against HOXB7 with siNC as a negative control were obtained from GenePharma (Shanghai, China). and sequences of the HOXB7 siRNA including siRNA-1: 5′-GCUAUUGUAAGGUCUUUGUTT, 5′-ACAAAGACCUUACAAUAGCTT; siRNA-2: 5′-CCCUUUGAGCAGAACCUCUTT, 5′-AGAGGUUCUGCUCAAAGGGTT; The final concentration of 100 nM siRNA or siNC pre-coated with Lipofectamine 3000 (Invitrogen, USA) were used for transfection.

Lentiviral vectors encoding the short hairpin RNAs (shRNAs) that target HOXB7 with the sequence of and a scramble shRNA were purchased from GenePharma (Shanghai, China). Transfection processes were conducted according to the instructions provided by the manufacturer. To generate the stable cell line, The transduced cells were then selected in culture medium containing puromycin (5.0 µg/ml).

### RNA extraction and quantitative real-time PCR

Total RNA was extracted by trizol reagent according to the manufacturer’s protocol. Two micrograms of RNA was reverse-transcribed into cDNAs and subjected to PCR reactions using the Prime-ScriptTM RT-PCR kit (Takara). Primers used for real-time PCR were as follows: HOXB7, forward: 5′-TTCCCAGAACAAACTTCTTGTGC-3′; reverse: 5′-GCATGTTGAAGGAACTCGGCT-3′. 18sRNA, forward: 5′-ACACGGACAGGATTGACAGA-3′; reverse: 5′-GGACATCTAAGGGCATCACA-3′. All of the determinations were performed in duplicate. The relative expression of HOXB7 mRNA was normalized to the expression level of 18sRNA mRNA using the 2−ΔCt method.

### Western blot analysis

Total protein was extracted from tumor cells. Equal amounts of protein were loaded onto a 10 % SDS-PAGE and electrophoresed and transferred onto PVDF membrane for 60–90 min based on the molecular weight of the target protein. After the membranes were blocked with 5 % non-fat milk, they were incubated overnight at 4 °C with the following primary antibodies: HOXB7 (1:1000, H00003217-M03, Abnova), vimentin (1:2000, #5741, Cell signaling, USA), E-cadherin (1:2000, #3195, Cell signaling), N-cadherin (1:1000, #13,116, Cell signaling), CD44 (1:1000, #6024-1-Ig, Proteintech, USA), CD133 (1:1000, #18470-1-AP, proteintech), ALDH1A1(1:1000, #15910-1-AP, proteintech), Bmi1 (1:1000, Bmi1, Cell signaling), SOX2 (1:1000, #4900, Cell signaling) and GAPDH (1:5000, MB001, Bioworld, China) followed by incubation with horseradish peroxidase (HRP)-conjugated secondary antibodies. Immunoreactive bands on the blots were detected by ECL chemiluminescence kit (Bio-Rad, USA).

### CCK‑8 and BrdU assay

Cell Counting Kit-8 (CCK8) assay (Cell Counting Kit-8, Dojindo, Japan) was used to measure cell viability. After transfection, cells were placed in 96-well plates at a density of 2 × 10^3^ cells per well; the absorbance values were detected 0 to 3 days after transfection. 10 µl of CCK-8 solution was added daily to each well flled in the 96-well plates and incubated for another 2 h. Then, a microplate reader (Multiskan MK3, Thermo, USA) was used to measure the absorbance at 450 nm. For BrdU assay, 2 × 10^5^ cells were inoculated into a 6-well culture plate (with a cover slip placed inside) for 24 h, then incubated with 1.0 mg/ml BrdU solution (Applied Biosystems, USA) for 4 h. The culture solution was then discarded, followed by cell fixation in methanol for 10 min and cell staining in diamidine phenyl indoles (DAPI; Thermo Fisher Scientific). BrdU-positive cells were arbitrarily counted in three visual fields through the microscope.

### Tumorsphere formation assay

In total, 2 × 10^4^/ml cells were plated onto ultra-low-attachment plates (NUNC, Thermo, USA) and cultured in DMEM/F12 serum-free medium (Invitrogen, USA) supplemented with B27 (BD Bioscience), 10 ng/ml b-fibroblast growth factor (bFGF, BD Bioscience, USA), 20 ng/ml epidermal growth factor insulin (BD Bioscience). The number of spheres was captured and counted under an inverted microscope after 10 days.

### Cell apoptosis assessed by flow‑cytometric assay

Cells were harvested and resuspended in 500 µl of binding buffer, and stained with Annexin V-FITC/PI Apoptosis kit (BD Biosciences). Apoptosis percentages were then detected using a FACSC aliber flow cytometer (BD Biosciences) and analyzed by Flowjo V10.1.

### In vitro cell invasion and wound healing assay

Cell invasion was determined by a Matrigel transwell invasion assay. Cells (1 × 10^5^/well) were suspended in 200 µl of serum-free DMEM and added to the upper chamber of an insert (8 μm pore size, Millipore, Germany) coated with Matrigel (BD Biosciences, USA). And Then, 600 µl DMEM with 10 % FBS were added to the lower chamber. For wound healing assay, Cells (2 × 10^5^ cells/well.) were seeded onto a 6-well plate overnight. The confluent monolayers were scratched using sterile pipette tips and washed with phosphatebuffered saline (PBS) 3 times to remove detached cells. The wounds were photographed at 0, 6, 12 and 24 h as indicated.

### Immunofluorescence assay

Cells were seeded on glass coverslips 18 h prior to the experiment and fixed with 4 % paraformaldehyde and washed thoroughly with PBS. Then permeabilized in 0.1 % Triton X-100 (Sigma-Aldrich). The cells were washed with PBS and blocked with 3 % bovine serum albumin (BSA) for 30 min at 37 °C. Then, incubation with primary antibodies against HOXB7 overnight. Cell were followed by incubation with secondary antibodies for 1 h. DAPI was used to counterstain DNA. Immunofluorescence images for HOXB7 were viewed with Zeiss fluorescence microscope.

### Immunohistochemical staining and scoring

Paraffin-embedded tissue samples from HNSCC patients were sliced into 4-µm-thick sections. Tumor tissues from mice were also sectioned at a 4-µm thickness using a thin semiautomatic microtome. All sections were deparaffinized in xylene and rehydrated in a series of graded alcohol dilutions. Antigen retrieval was performed by heating in a microwave oven. Then, the sections were incubated with 3 % H2O2 for 10 min followed by 10 % normal goat serum for 15 min at room temperature to block endogenous peroxidases and non-specific antigens. Histological sections were immunostained overnight at 4 °C using the following primary antibodies: anti-HOXB7 antibody (1:200, #H00003217-M03, Abnova), anti-CD133 antibody, anti-CD44 antibody and anti-Ki67 antibody. Negative controls (only PBS incubation) were included in each staining run. Immunoreactivity in each slide was semi-quantitatively evaluated according to staining intensity and distribution and the immunoreactive score was calculated as intensity score × proportion score. Intensity score was defined as 0, negative; 1, weak; 2, moderate; 3, strong, while the proportion score was evaluated by two independent pathologists via counting positive nucleus with 0, negative; 1, < 10 %; 2, 11–50 %; 3, 51–80 %; 4, > 80 % positive cells. The immunoreactivity of each slide was categorized into three subgroups according to the final score: 0, negative; 1–4, low expression; ≥4, high expression.

### HNSCC xenograft animal model

All experiments involving animal subjects were in accordance with the institutional animal welfare guidelines and approved by Institutional Animal Care and Use Committee of Nanjing Medical University. Six-week-old female nude mice were purchased from Model Animal Research Center of Nanjing Medical University and maintained in the specific pathologic-free animal facility. 2 × 10^6^ cells of Fadu in 100 µl PBS then subcutaneously injected into both flanks of each animal (6 animals per experimental group). Sizes of tumors and were measured every 3 days when tumour masses were identified. Tumor volume = [(length) × (width) × (width)]/2. All the mice involved was sacrificed by intraperitoneal injection of a deadly dose of pentobarbital sodium (150 mg/kg) at the 31th day after tumor cell injection. When the vital signs of mice disappeared, the end-point tumor was dissected, weighed and recorded.

### Bioinformatics analysis of HOXB7 from public databases data sources

The RNA sequencing and clinical data were obtained from The Cancer Genome Atlas (TCGA) database. The microarray datasets: GSE6631, GSE12452, GSE23036, GSE25099, GSE30784, GSE42743 and GSE9844 were downloaded from the Gene expression Omnibus (GEO) database.

### Functional enrichment of HOXB7 co-expression genes

In this study, we used the Pearson correlation coefficient (r) to screen and identify HOXB7-related genes with a *P* < 0.05 and |r| > 0.3 were identified as HOXB7-related genes. The biological functions of these HOXB7 co-expression genes were comprehensively detected by GO enrichment and Kyoto encyclopedia of genes and genomes (KEGG) pathway analysis. In addition, the protein–protein interactions (PPIs) among all HOXB7 co-expression genes were obtained by Search Tool for the Retrieval of Interacting Genes (STRING; http://string-db.org) and the network was constructed with the Cytoscape 3.7.0 software.

### Survival analysis and small molecule targeted drugs screening of HOXB7 in HNSCC

The Connectivity Map (CMap) (http://www.broad.mit.edu/cmap)was used to identify potential small molecule targeted drugs for HOXB7 in HNSCC. Those small molecule drugs with mean connective score < − 0.2 and P < 0.05 were recognized as the possible therapeutic drugs of HOXB7 in HNSCC.

### Statistical analysis

Data between two groups were examined using a two-tailed paired Student’s t-test or or ANOVA (Bonferroni post hoc test). The Chi-squared test was applied to assess the correlation between HOXB7 expression and various clinicopathological parameters. Survival data were used to establish Kaplan–Meier curves, and the differences among the groups were analyzed by the log rank test. Univariate and multivariate Cox-regression analysis were employed to determine prognostic factors associated with survival. Two-tailed P values < 0.05 were considered as statistically significance. All statistical analyses were performed by GraphPad Prism 7 or R (4.0.2).

## Results

### Aberrant upregulation of HOXB7 mRNA in HNSCC via bioinformatics analyses

**Fig. 1:**
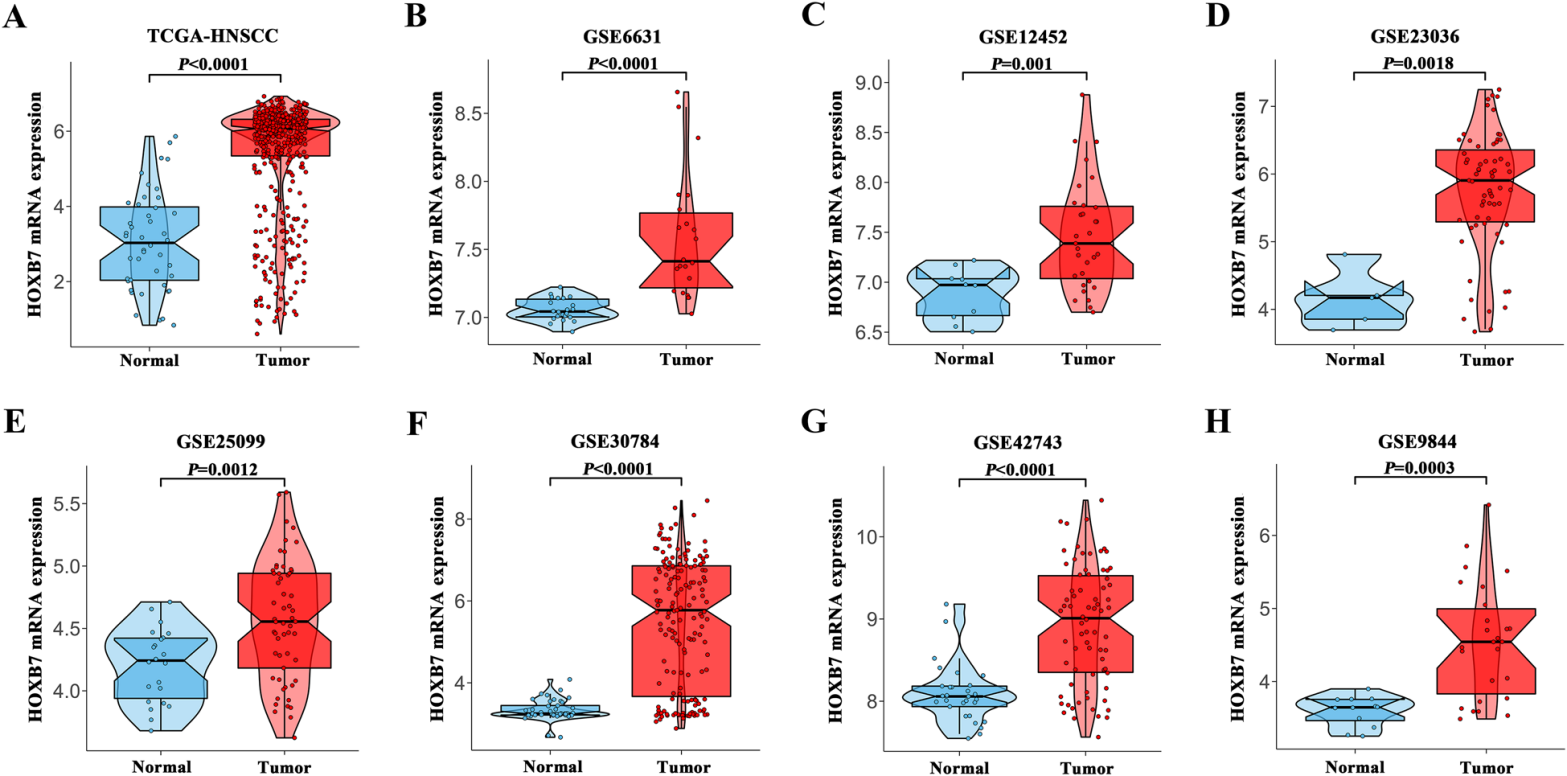
Overexpression of HOXB7 mRNA in HNSCC datasets. **A–H** The mRNA levels of HOXB7 (normalized and log2-transformed) were compared between HNSCC samples and normal counterparts in TCGA-HNSC (**A**), GSE6631 (**B**), GSE12452 (**C**), GSE23036 (**D**), GSE25099 (**E**), GSE30784 (**F**), GSE42743 (**G**) and GSE9844 (**H**) datasets. The original data were retrieved from GEO and TCGA databases. *HNSCC* head neck squamous cell carcinoma, *HOXB7* Homeobox B7

The workflow of our study and data analytic pipeline was illustrated in Additional file 2: Fig. S2. Firstly, we used publicly available sets such as TCGA (The Cancer Genome Atlas) and GEO (Gene Expression Omnibus) datasets to analyze relevant information on HOXB7 expression patterns. Integration and analysis of the TCGA-HNSC cohort (502 cases) data showed that HOXB7 mRNA was obviously up-regulated in the TCGA-HNSC specimens compared with the normal counterpart (44 cases) using TCGA dataset (Fig. 1A). Moreover, seven independent HNSCC patients cohorts from GEO database such as GSE6631, GSE12452, GSE23036, GSE25099, GSE30784, GSE42743 and GSE9844 cohorts were identified and utilized to measure HOXB7 mRNA expression. As shown in Fig. 1B–H, significantly higher abundance of HOXB7 mRNA was observed in HNSCC samples compared to their non-tumor counterparts. Multiple evidence has indicated that HOXB7 was aberrantly overexpressed in multiple cancers and associated with unfavorable prognosis [21, 28].

### Overexpression of HOXB7 correlates with aggressive clinicopathological parameters in HNSCC

**Fig. 2:**
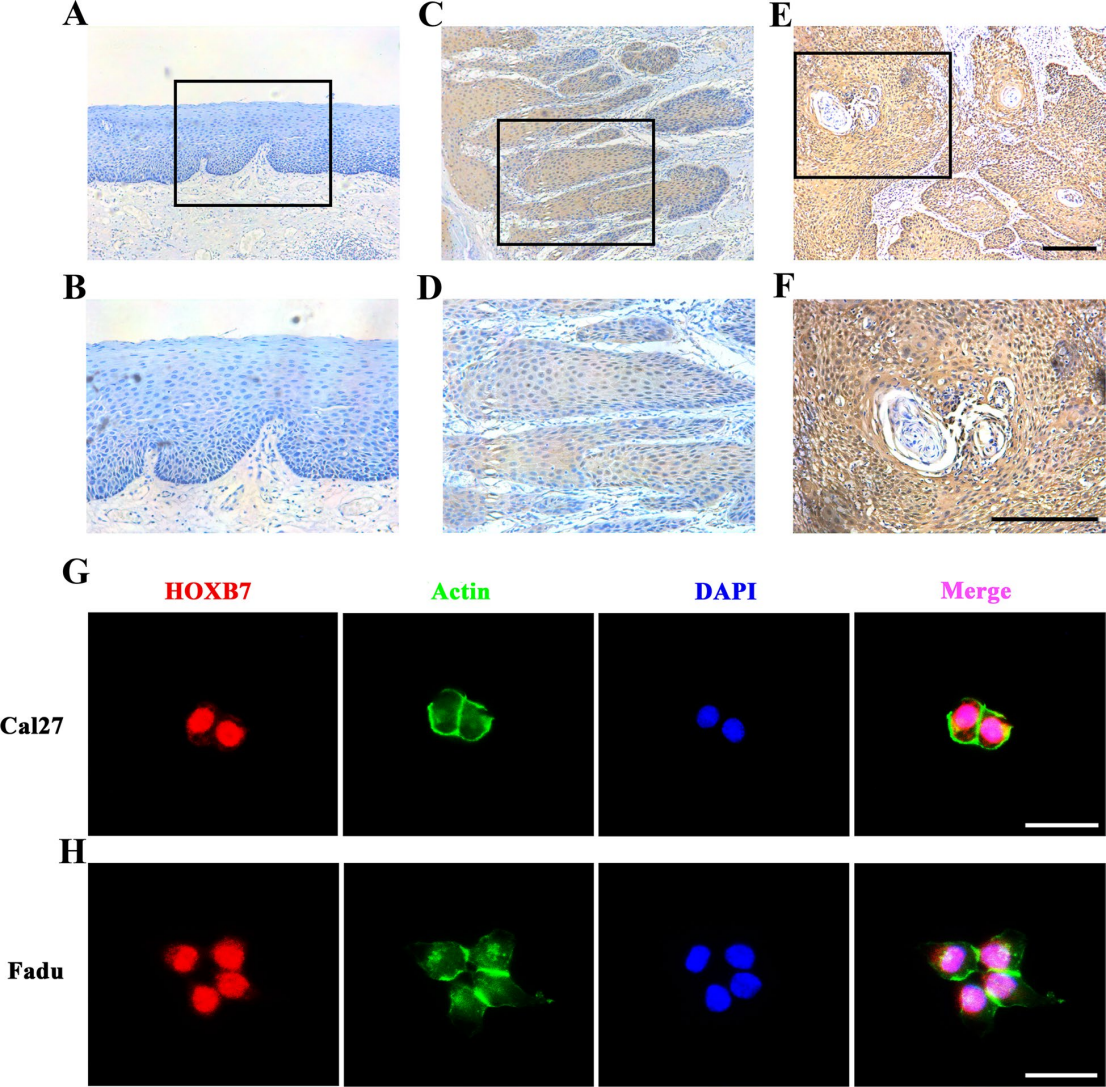
HOXB7 protein expression and location in human HNSCC samples and cell lines were detected by immunohistochemical and immunofluorescence staining, respectively . **A–F** The immunohistochemical staining of human HNSCC samples. Representative negative staining of HOXB7 in normal oral epithelial (**A, B**); Representative low expression of HOXB7 in primary human HNSCC sample (**C, D**); Representative high expression of HOXB7 in primary human HNSCC sample (**E, F**). The areas marked by black box in the **A, C, E** images (upper panel) were shown in larger magnification as **B, D, F** images (lower panel), respectively. Scale bar: 100 μm. **G, H**: The immunofluorescence staining of HOXB7 in Cal27 and Fadu cells. HOXB7 was predominantly identifified in nucleus and rarely in cytoplasm in Cal27 (**G**) and Fadu (**H**). Nuclei are counterstained with DAPI. Scale bar: 100 μm

To further find out the expression pattern of HOXB7 in HNSCC specimens, we next performed immunohistochemical staining of HOXB7 from our clinical cohort which contains 119 primary HNSCC samples. As displayed in Fig. 2A–F, HOXB7 showed positive staining mainly in the nucleus in cancerous paraffin sections, whereas weak/negative staining was identified in the normal mucosa and the stroma of HNSCC. Based on our IHC-scoring system, HOXB7 expression in HNSCC/normal mucosa was classified, in HNSCC (high, n = 71 versus low, n = 48) and in normal clinical specimens (high, n = 4 versus low, n = 10 versus negative, n = 12). Therefore, these data confirmed that the HOXB7 protein was highly expressed in HNSCC (P < 0.001, Chi-square test). Then, detailed clinical data of this cohort is given in Table 1 (52 females versus 67males, average 61.49 years). The patient’s latest follow-up time ranged from 3 to 83 months). The correlation between HOXB7 expression and clinicopathological parameters was displayed in Table 1. It is clear that there was no obvious association between HOXB7 expression and patients’ age, drinking and heavy tobacco usage, tumor size and gender. Notably, HOXB7 expression was positively associated with cervical lymph nodes metastasis, pathological grade and clinical stage with *P* value 0.0195, 0.03 and 0.0152, respectively, which indicated HOXB7 may be implicated in the initiation and progression of HNSCC. Moreover, immunofluorescence was performed to visualize the subcellular distribution of HOXB7 protein in both Fadu and Cal27 cells. As data showed in Fig. 2G-H, HOXB7 was mainly enriched in the nucleus but much less in the cytoplasm in both cell lines which consistent with the location of the transcriptional factor.

**Table 1:** The associations between HOXB7 expression and multiple clinicopathological parameters in HNSCC Samples

Clinicopathological parameters	Samples	HOXB7		P values
		High	Low	
Gender		71	48	0.4455
Male	67	42	25	
Female	52	29	23	
Age				0.2631
≤ 60	57	37	20	
> 60	62	34	28	
Smoking				0.3508
No	81	46	35	
Yes	38	25	13	
Alcohol				0.2404
No	90	51	39	
Yes	29	20	9	
Pathological grade				**0.03**
I	65	33	32	
II–III	54	38	16	
Clinical stage				**0.0152**
I–II	51	24	27	
III–IV	68	47	21	
Tumor size				0.3508
T1–T2	81	46	35	
T3–T4	38	25	13	
Cervical node metastasis				**0.0195**
N(0)	69	35	34	
N(+)	50	36	14	

Bold indicates statistical significance, with P values less than 0.05

### **HOXB7 aberrant overexpression associated with reduced survival in HNSCC patients**

**Fig. 3:**
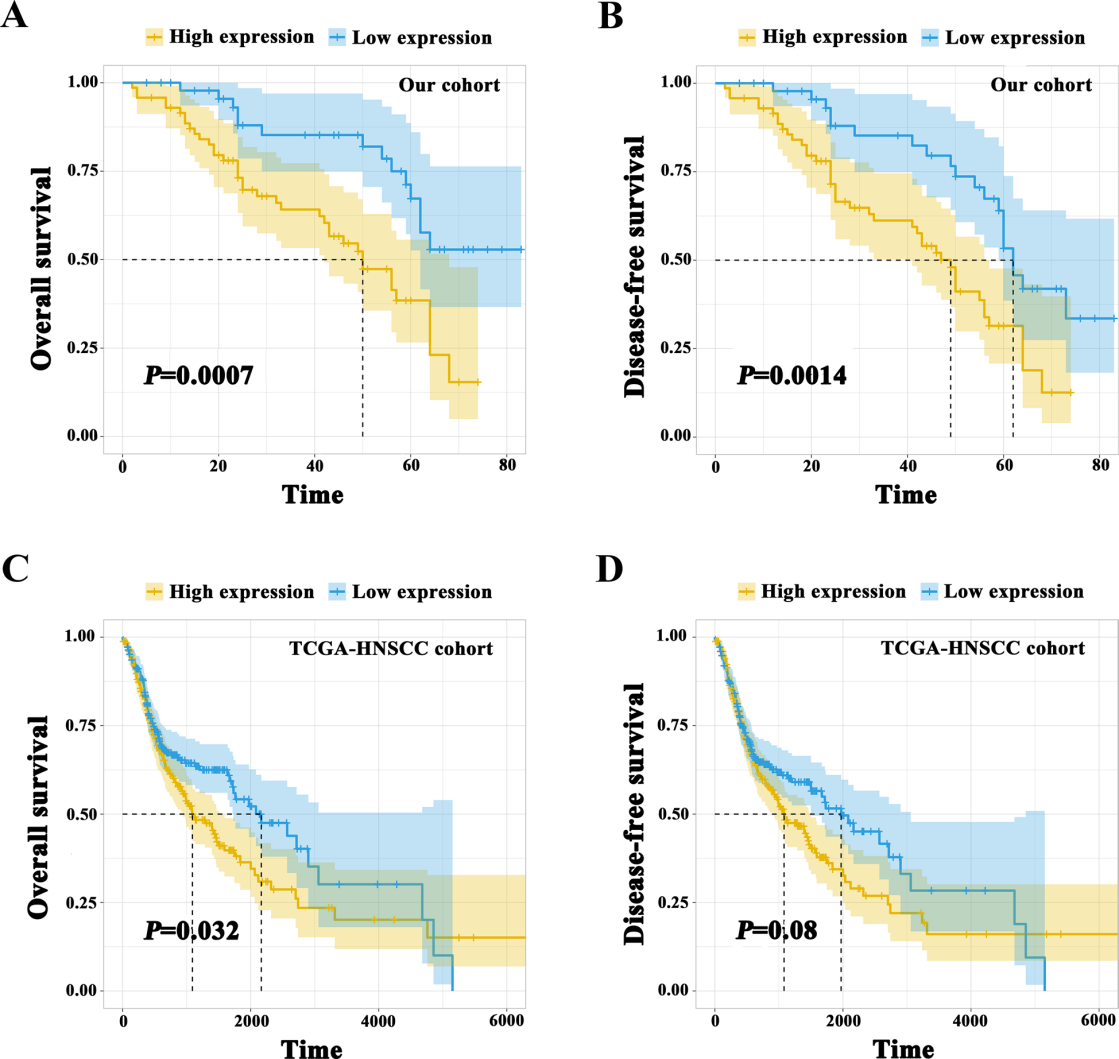
High HOXB7 expression positively associates with reduced overall survival and disease-free survival rates in HNSCC patients. **A, B** Overall survival (**A**) and disease-free survival (**B**) analyses of HNSCC patients with high or low expression of HOXB7 were estimated by IHC score; IHC, immunohistochemical staining score. **C, D** Overall survival (**C**) and diseases-free survival analyses (**D**) of TCGA-HNSCC patients with high or low expression of HOXB7 mRNA (median value as cutoff) were estimated by Kaplan-Meier method and compared with Log-rank test

To explore the association between HOXB7 expression and prognosis of patients with HNSCC, we attempted to evaluate the relationship between HOXB7 protein expression and clinical outcomes. According to the last follow-up data, 57 (47.9 %) patients were still disease-freely alive, 11 (9.2 %) survival with cervical nodal metastasis and/or local recurrences, whereas 51 (42.9 %) patients died of post-surgical relapse, cancer metastases or other diseases. Furthermore, the Kaplan-Meier analysis showed that patients with high HOXB7 abundance had obviously shorter overall-survival and disease-free survival than patients with low high HOXB7 abundance (Log-rank, *P* = 0.0007, 0.0014. Figure 3A, B). Moreover, the similar conclusion from TCGA-HNSC cohort showed that the overall survival proportions in HOXB7 high expression groups was also significantly lower than those in HOXB7 low expression groups (Log-rank, *P* = 0.032, Fig. 3C). In addition, we observed HOXB7 high expression groups have lower disease-free survival proportions than HOXB7 low expression groups while it was no statistical differences. (Log-rank, *P* = 0.08, Fig. 3D).

**Fig. 4:**
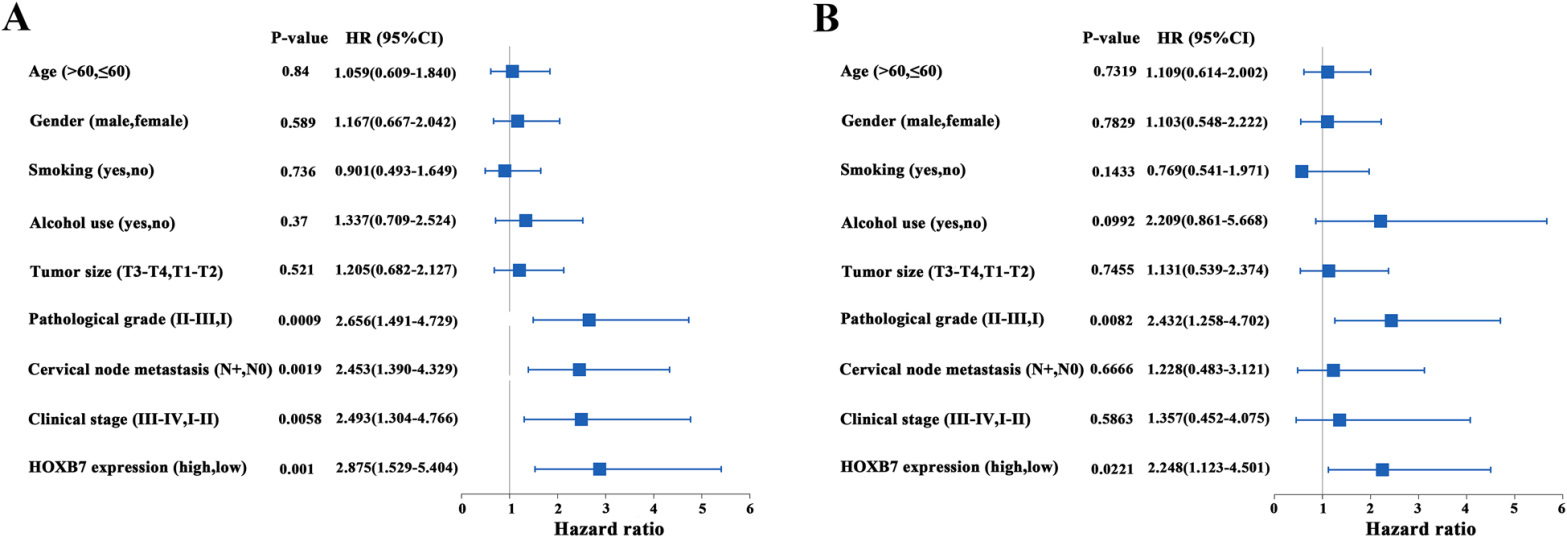
Univariate and multivariate Cox-regression analyses of HOXB7 and clinicopathological parameters associated with overall survival for patients with primary HNSCC. **A**: Univariate Cox-regression analyses revealed that HOXB7 expression, pathological grade, cervical nodal metastasis and clinical stage were significantly associated with overall survival. **B**: Multivariate Cox-regression analyses indicated HOXB7 expression were found to be an independent prognostic marker for the overall survival of patients with HNSCC

Moreover, we applied both univariate and multivariate Cox-regression analysis to further evaluate the prognostic value of HOXB7 expression in HNSCC. Univariate Cox-regression analysis showed pathological grade [hazard ratio (HR), 2.656; 95 % confidence interval (95 % CI), 1.491–4.729; *P* = 0.0009], cervical nodal metastasis[HR, 2.453; 95 % CI 1.39–4.329; *P* = 0.0019], clinical stage [HR, 2.493; 95 % CI, 1.304–4.766; *P* = 0.0058] and HOXB7 expression [HR, 2.875; 95 % CI, 1.529–5.404; *P* = 0.0010] significantly associated with overall survival, while other clinicopathological variables did not reach the statistical significance as indicated in Fig. 4A. Multivariate Cox-regression analysis showed the HOXB7 expression could be used as an independent prognostic factor affecting patient overall survival (HR, 2.248; 95 % CI, 1.123–4.501; *P* = 0.0221), (Fig. 4B) after adjusting some well-established prognostic factors like clinical stage, pathological grade, tumor size and cervical node metastasis.

### **HOXB7 depletion impairs proliferation and triggers apoptosis in HNSCC cells in vitro**

**Fig. 5:**
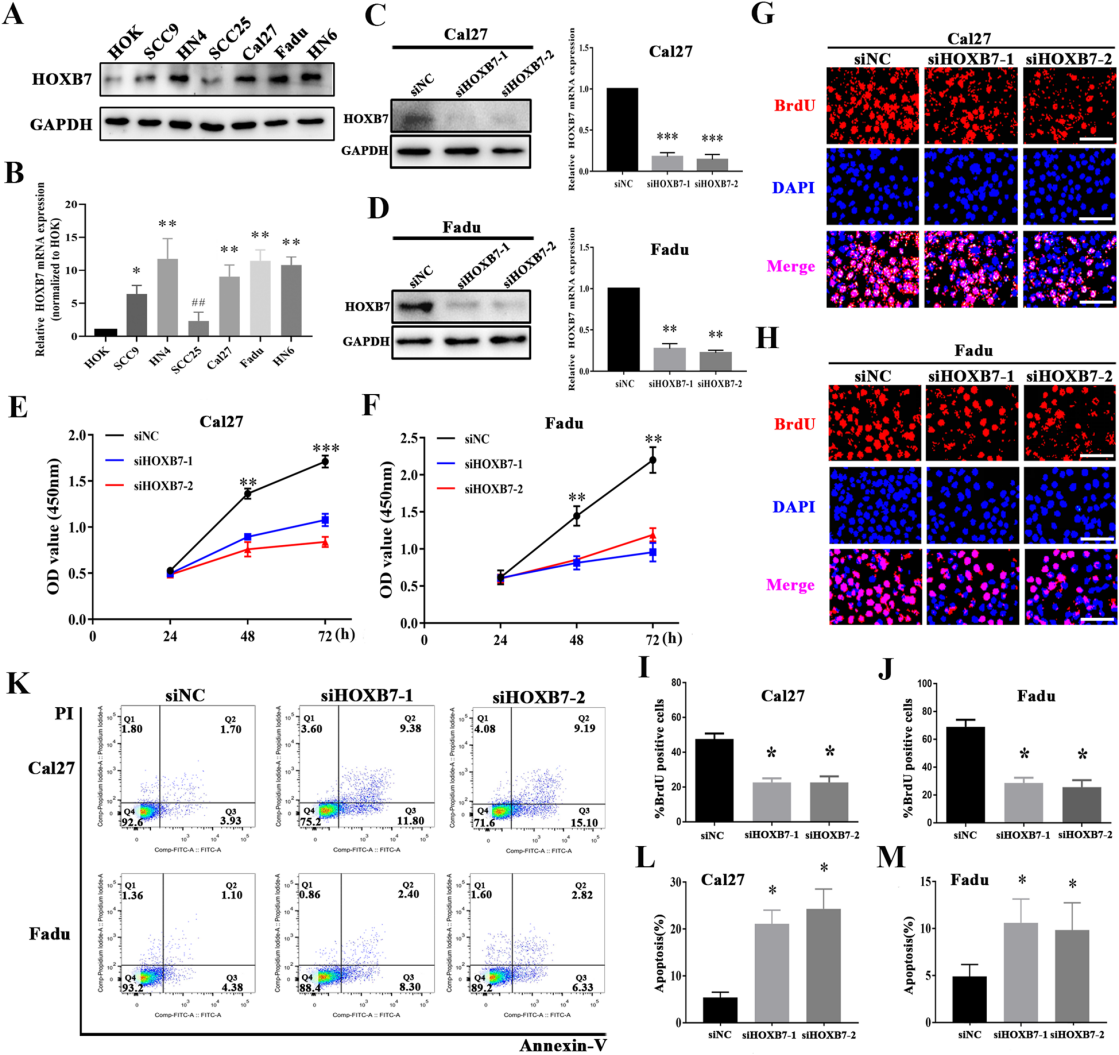
HOXB7 knockdown inhibits cell proliferation and triggers apoptosis in HNSCC cells in vitro. **A, B**: Endogenous HOXB7 protein and mRNA expression were measured in a panel of HNSCC cell lines as compared to normal human oral keratinocytes (HOK); **C, D**: Endogenous HOXB7 protein and mRNA were efficiently silenced by two siRNAs (siHOXB7-1, siHOXB7-2) in Fadu and Cal27 cells. Non-targeting siRNA was utilized as negative control (siNC); **E–J**: Cell proliferation was remarkably suppressed when endogenous HOXB7 was silenced as measured by CCK8 (**E, F**) and BrdU assays (**G–J**); **I–M**: Increased percentages of cell undergoing apoptosis were evident following HOXB7 knockdown as assayed by Annexin V-PI staining. Scale bar: 100 μm. Data shown from three independent experiments, *P < 0.05, **P < 0.01, ANOVA analyses

Considering that our clinical results supported a potential pro-tumorigenic role of HOXB7 in HNSCC. However, its oncogenic roles in HNSCC initiation and progression still uncovered yet. To address this, we first measured the expression abundance of HOXB7 in a panel of HNSCC cell lines and found that HOXB7 mRNA and protein were significantly overexpressed in all HNSCC cell lines examined compared to immortalized oral epithelial cell (HOK) (Fig. 5A, B). Due to the relatively higher endogenous HOXB7 in Cal27 and Fadu cells, we next selected them for knockdown experiments. Two independent siRNAs targeting human-HOXB7 (siHOXB7-1, siHOXB7-2) were introduced into Fadu and Cal27 cells to detect the changes of HOXB7 protein and mRNA expression and alterations of cell phenotypes. As shown in Fig. 5C, D, the expression of HOXB7 protein and mRNA decreased significantly after transfection with siHOXB7, which confirmed the effectiveness of our loss-of-function assay. And then, we detected the phenotypic changes associated with HOXB7 knockdown. Following HOXB7 downregulated, the proliferation and viability of both Fadu and Cal27 cells were obviously impaired as measured by CCK-8 assays (Fig. 5E, F) and BrdU assays (Fig. 5G–J). The results showed that the ability of cell proliferation and survival decreased significantly in siHOXB7-treated cells. Moreover, Annexin V-PI Flow cytometric experiment showed an obviously increased in apoptosis rate of siHOXB7-treated cells. The apoptosis ratios were increased from 5.2 to 22.6 % in Cal27 and from 3.8 to 13.7 % in Fadu, respectively (Fig. 5K–M).

### HOXB7 knockdown inhibited migration and invasion in HNSCC cells

**Fig. 6:**
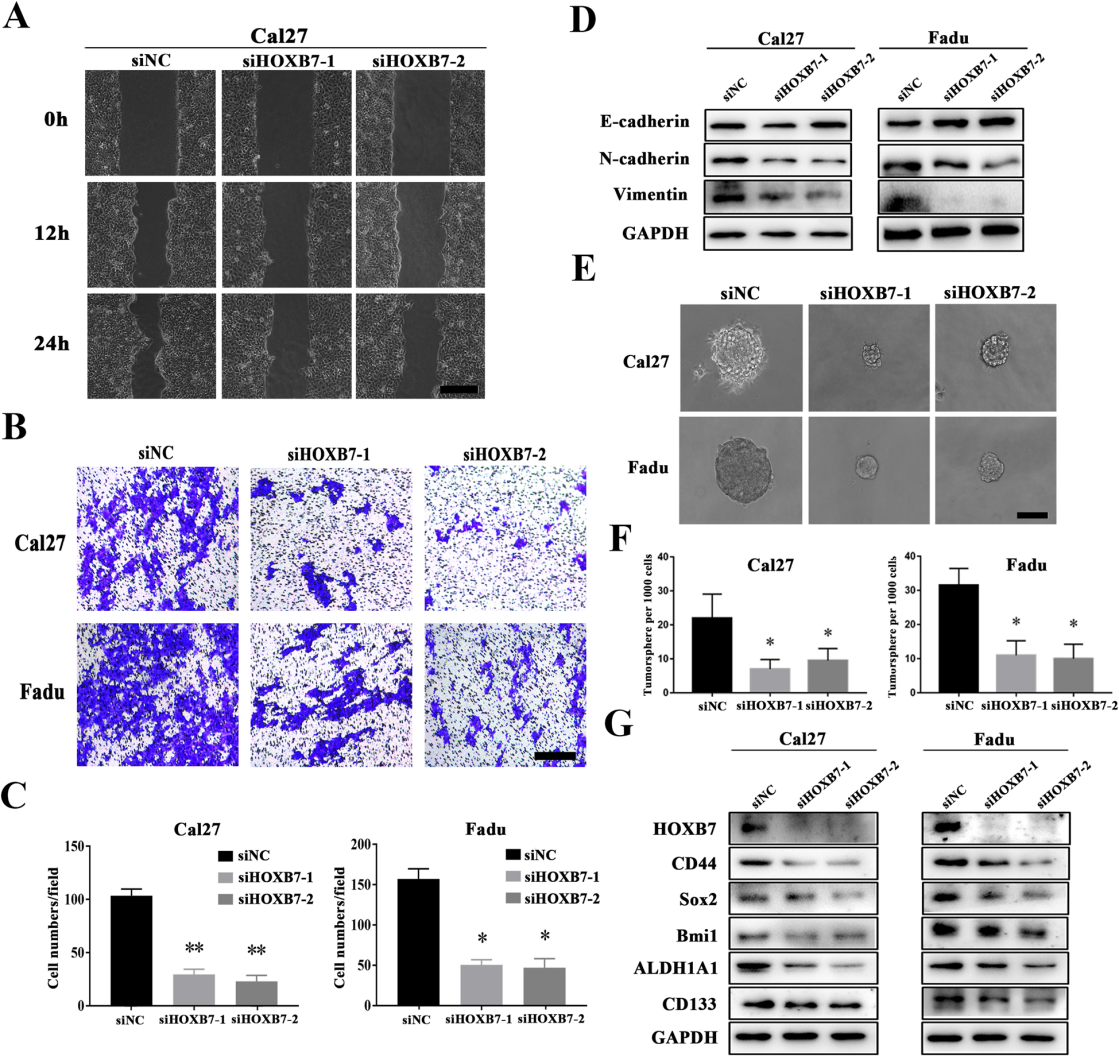
Knockdown of HOXB7 inhibited the migration and invasion and the expression of CSCs-related makers in Cal27 and Fadu cells. **A–C**: The cell motilities and invasion were remarkably diminished after HOXB7 knockdown as gauged by wound healing (**A**) and transwell-invasion assay (**B, C**). Measurements of wound healing was performed at 12 and 24 h after cell scratching while measurements of transwell assays were done at 12 h after cell seeding; **D**: The abundance of EMT-related markers E-cadherin, N-cadherin and vimentin were measured by western blot (WB) in the Cal27 and Fadu cells following HOXB7 knockdown; **E, F**: The capability of tumorsphere formation was markedly reduced in siHOXB7-transfected cells relative to control cells (**E**). Quantification of primary and secondary tumorspheres culture were shown (**F**); **G**: The abundances of cancer stem cells (CSCs) - related makers were measured by western blot (WB) in the Cal27 and Fadu cells following HOXB7 knockdown. Scale bar = 100 μm. Data showed here are mean ± SD from three independent experiments; ANOVA analyses. CSCs, cancer stem cells; SD, standard deviation. *P < 0.05; **P < 0.01

Our clinical results suggested that HOXB7 protein expression was associated with cervical lymph node metastasis in HNSCC patients. To further determine whether HOXB7 participates in the regulation of migration and invasion process in HNSCC cells, wound healing and transwell experiments were executed to examine the migration and invasion ability of HOXB7 knockdown cells, respectively. Transwell assays showed that knockdown of HOXB7 weakened the invasion and migration ability of Cal27 and Fadu cells (Fig. 6B, C). Furthermore, wound healing assay showed that knockdown of HOXB7 inhibited the migration of Cal27 and Fadu cells (Fig. 6A). To further validate the relationship between HOXB7 and migration and invasion of HNSCC cells, we detected the protein level of EMT/metastasis-associated markers such as N-cadherin, E-cadherin, and Vimentin. And found that HOXB7 knockdown reduced the expression of N-cadherin and Vimentin, and increased the expression of E-cadherin (Fig. 6D). Our results implied that HOXB7 might regulate the migration and invasion of HNSCC cells by promoting EMT. In addition, tumorsphere formation assay performed and displayed in Fig. 6E, F, comparing with siNC group, tumorsphere formatting ability of siHOXB7-treated cells was pronouncedly impaired in Cal27 and Fadu cells. Proliferation and sphere formation are characteristics of cancer stem cells. To determine whether HOXB7 regulates the stemness of HNSCC cells, we detected the expression of cancer stem cell (CSCs) related markers. And found that knockdown of HOXB7 in Cal27 and Fadu cells inhibited the expression of ALDH1A1, CD44, CD133, Bmi1 and SOX2 (Fig. 6G). These results suggested that HOXB7 maybe regulate the malignant progression of HNSCC cells by modulating stem-related characteristics.

### **HOXB7 knockdown impairs tumour growth in an HNSCC xenograft model**

**Fig. 7:**
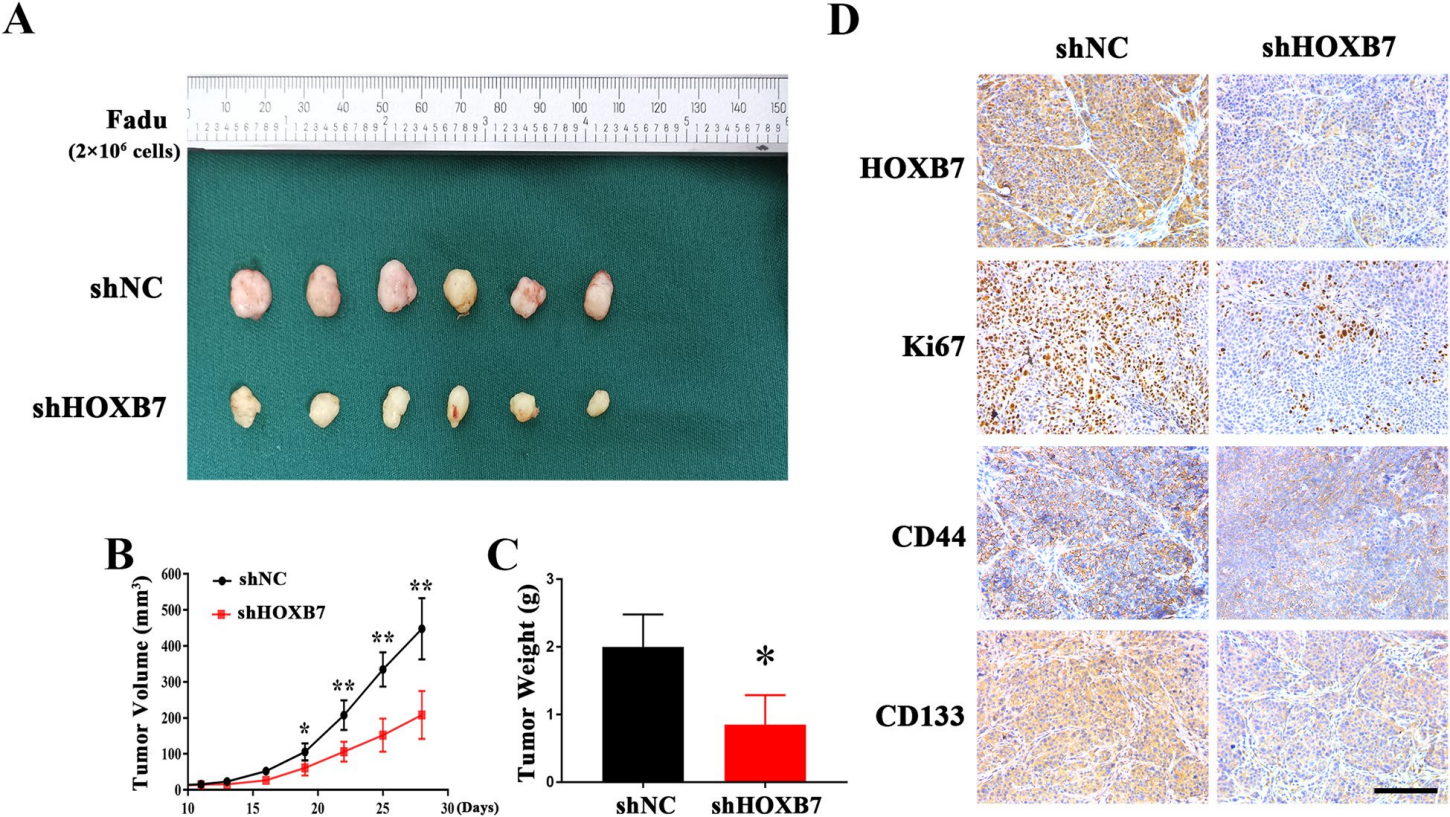
HOXB7 depletion impaired tumor growth in a HNSCC xenograft model. **A, B**: Tumour volume was monitored in xenograft samples derived from Fad cells with stable HOXB7 knockdown or controls; **C**: Final weight of tumour masses harvested from derived from Fadu cells with stable HOXB7 knockdown or controls was compared. **D**: The marker indicative of HOXB7 knockdown and proliferative marker Ki67 and CSCs markers CD44 and CD133 were determined by immunohistochemical staining in xenograft samples derived from Fadu cells with stable HOXB7 knockdown or controls. Scale bar: 100 μm. Representative images are shown. *P < 0.05, **P < 0.01, Student’s *t-*test

To further confirm the carcinogenicity of HOXB7 in vivo, we established a HNSCC xenograft model by inoculating Fadu cells stably depleted of HOXB7 into the left abdomen of nude mice and then monitoring the occurrence and growth of tumors after cell injection. As shown in Fig. 7A–C, compared with control cells, tumor growth in xenograft samples formed by siHOXB7-treated cells was impaired, and tumor volume and weight were significantly reduced. However, we failed to identify the difference of tumour incidence between two types of grafts, although the delay of tumour onset in grafts of siHOXB7-treated cells was observed. Immunohistochemical staining of tumor samples showed that the positive staining of stem cell markers in HOXB7 knockdown cell samples was significantly reduced compared with the control (Fig. 7D). In addition, the number of Ki67-positive cells in tumor samples derived from HOXB7 knockdown cells was significantly reduced compared to samples formed from control cells (Fig. 7D). Together, these findings suggested that HOXB7 was critically involved in tumour overgrowth of HNSCC.

### The biological functions analysis of HOXB7 in HNSCC

**Fig. 8:**
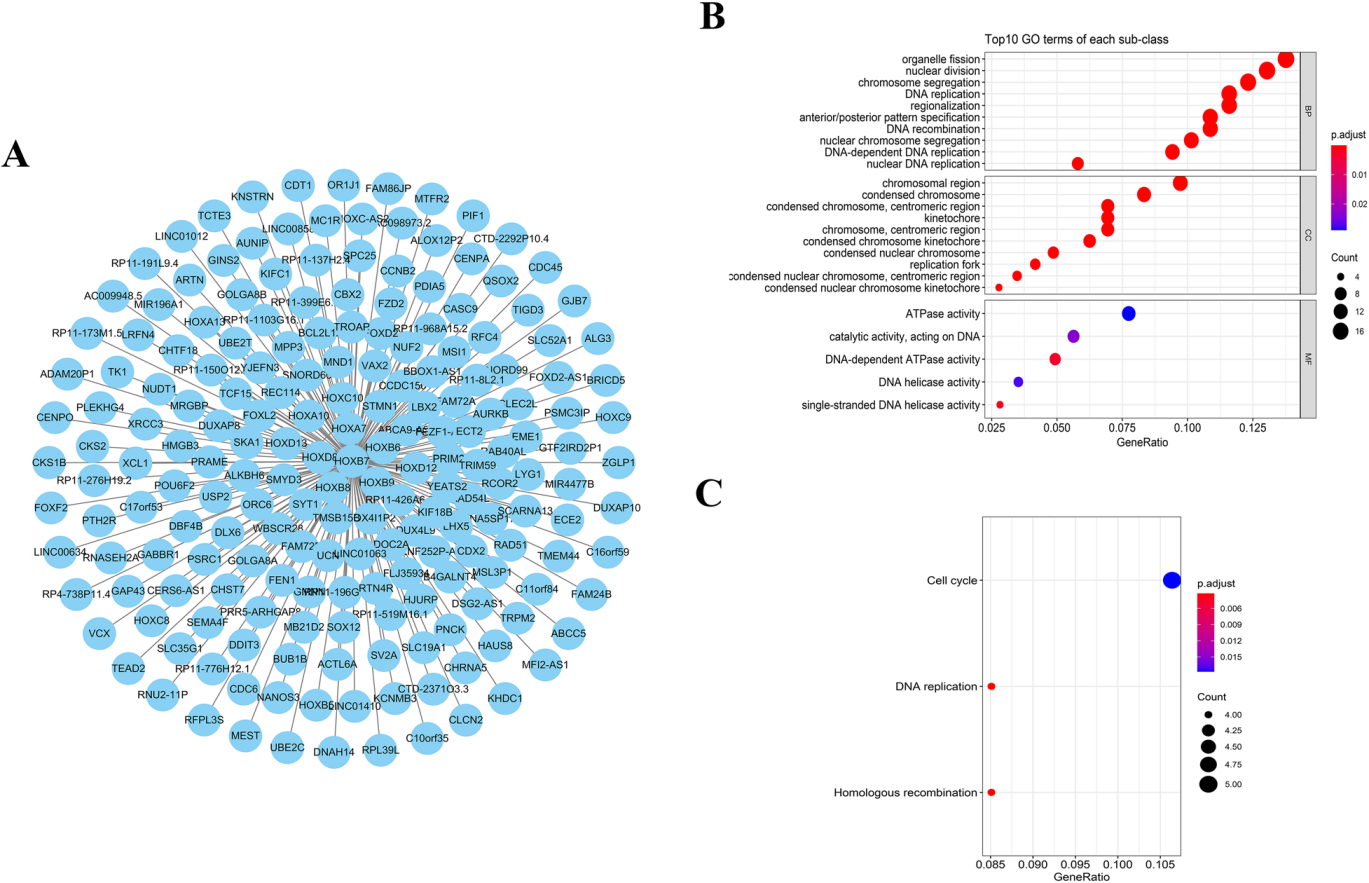
Bioinformatics analysis was carried out to investigate HOXB7 expression related genes. **A**: Screen and identify HOXB7-related genes with a *P* < 0.05 and |r| > 0.3 were identified as HOXB7-related genes by using the Pearson correlation coefficient (r); **B, C**: The biological functions of these HOXB7 co-expression genes were comprehensively detected by GO enrichment (**B**) and Kyoto encyclopedia of genes and genomes (KEGG) pathway analysis (**C**)

In order to investigate HOXB7 therapeutic potentiality, firstly, we conducted a genome-wide co-expression analysis of HOXB7 to explore pro-tumorigenic functions, and screened 198 HOXB7 related genes and all genes were positively correlated with HOXB7 **(**Fig. 8A**)**. Then we carried out GO analysis for these co-expressed genes and these HOXB7 related genes were participated in Organelle fission, Nuclear division, DNA replication, Chromosome region, ATPase activity and catalytic activity, Acting on DNA **(**Fig. 8B). KEGG analysis showed that HOXB7 related genes enriched in multiple pathways such as Cell cycle, DNA replication and Homologous recombination **(**Fig. 8C**)**. These results suggest that a widespread impact of HOXB7 on the global transcriptome regulation.

To further explore HOXB7 as a potential therapeutic target for HNSCC, we performed a CMap analysis with the screening conditions under mean connective score < − 0.2 and *P* < 0.05, and finally screened three small molecule drugs potentially effective targeting HOXB7. They are NU-1025 (Mean connective score = − 0.560; *P* = 0.003), thiamine (Mean connective score = − 0.550; *P* = 0.038), vinburnine (Mean connective score = − 0.410; *P* = 0.018), and the chemical structures of which are shown in Additional file 1: Fig. 1. Those small molecule drugs with were recognized as the possible therapeutic drugs of HOXB7 in HNSCC.

## Discussion

Nowadays, it is still an urgent mission to find new biomarkers to provide new targets for the treatment of HNSCC especially under the condition of long-term survival rate of HNSCC patients has not improved significantly [29, 30]. HOXB7, a typical transcription regulator, encodes a homologous protein which is not only related to the normal development and differentiation of cells or organs, but also abnormal highly expressed in tumor cells or tissues, and participates in tumor initiated and progression. Up to now, a great quantity studies have revealed that the abnormal expression of HOXB7 was closely associated with malignant changes and poor prognosis of human tumors [16, 31, 32]. Here, combined with bioinformatics and clinical samples analysis, we found that HOXB7 protein and mRNA were significantly overexpressed in HNSCC, the high expression of which is closely related to clinical staging, lymph node metastasis and poor prognosis in patients. In addition, HOXB7 knockdown significantly impaired cell proliferation, migration and invasion and induced apoptosis in vitro and vivo. Moreover, HOXB7 RNAi-related silencing significantly inhibited the expression of CSCs related markers and reduced the ability of tumor spheres formation in HNSCC cells. Bioinformatics analysis supports that HOXB7 participates in the regulation of tumor malignant phenotype and can be used as a therapeutic target for small molecule compounds. Our results together others strongly suggested that as a newly hypothesized oncogene HOXB7 promotes HNSCC development and also a novel biomarker with clinical translation potential.

It is undisputed that deregulated of HOXB7 in multiple types of cancers [18, 28, 33]. For example, HOXB7 overexpression was reported in association with the clinical progression and poor outcome of patients with breast cancer [34]. Deregulation of HOXB7 expression in colorectal cancer predicted poor outcomes of patients [21]. Up-regulation of HOXB7 in pancreatic ductal adenocarcinoma was correlated with poor prognosis of patients [22]. Moreover, previous studies have shown that abnormally elevated HOXB7 expression was significantly associated with tumor size, cervical lymph node metastasis, malignancy, and etc. [32, 35]. Consistently, our data revealed that both bioinformatics analyses from multiple independent patient cohorts and immunohistochemistry in primary HNSCC samples revealed aberrant overexpression of HOXB7 in a large subset of patients examined. In addition, we found HOXB7 abnormal expression was closely related to cervical lymph node metastasis and clinical stage, while did not reach statistical significance with other pathological parameters. Moreover, Kaplan-Meier survival analysis showed HNSCC patients with higher HOXB7 expression have a poorer prognosis. Through univariate and multivariate Cox-regression analyses, we also identified that HOXB7 expression could be used as an independent prognostic indicator for HNSCC. Together, it unbiasedly characterized that HOXB7 could be independently used as a prognostic biomarker of HNSCC.

Accumulating evidence has shown that HOXB7 was crucial to promote cell proliferation and migration to participate in tumorigenesis and inhibit cell apoptosis [15, 16]. For example, in glioma, HOXB7 promoted invasion and migration by regulating the Wnt/β-catenin signaling pathway in glioma cells, and was significantly correlated with tumor lymph node metastasis or distant metastasis [32]. In osteosarcoma, HOXB7 silenced significantly inhibited proliferation and invasion of osteosarcoma cells [36]. Consistent with this, our findings in vitro loss of function test results showed that HOXB7 down-regulated inhibited the proliferation, migration and invasion and induced apoptosis of HNSCC cells. These findings were further confirmed by facts such as decreased ability of xenograft tumour growth after HOXB7 knockdown. Moreover, EMT has been increasingly recognized as a regulatory mechanism necessary for primary tumor cells to achieve migration and invasion [37]. Li et al. have reported that increased HOXB7 expression promoted EMT and metastasis in breast cancer [16]. Wu J et al. have reported that HOXB7 accelerated invasion and invasion process of gastric cancer cells by EMT [38]. Consistently, our results also indicated that HOXB7 promoted invasion and motility by facilitating EMT in HNSCC as evidenced by EMT marker changes upon HOXB7 depletion. In addition, bioinformatic analysis such as GO and KEGG from TCGA samples revealed the detailed tumor biology process which HOXB7 involved such as Organelle fission, Nuclear division, DNA replication, Chromosome region, ATPase activity and catalytic activity ,cell cycle all together ensure cancer cell survival and boosting proliferation. Previous studies have shown that HOXB7 enhanced DNA binding by PBX1 to mediate DNA replication [39]. Moreover, another study suggested that in colorectal cancer, HOXB7 accelerated G0-G1 to S-phase transition concomitantly with upregulation of cyclin D1 and downregulation of p27Kip1 in tumor cells. On the contrary, knockdown of HOXB7 caused G1-S-phase arrest, downregulation of cyclin D1 and upregulation of p27Kip1, which in part strengthened our data [21].

Previous studies had shown that HOXB7 overexpression in hepatocellular carcinoma boosting c-Myc and Slug transportation and activated MAPK-AKT pathway to facilitate stem-like properties and EMT process in hepatoma cells, which finally malignant progression [23]. In glioma, HOXB7 facilitated invasion and migration of tumor cells by activating the Wnt signaling, while obviously related to lymph node metastasis or distant metastasis [32]. In lung cancer, HOXB7 overexpression increases several iPSC markers and sustains the stemness of stem cell subpopulation by modulation of LIN28B [31]. However, these malignant phenotypes of tumor cells have been shown to be associated with a distinct subpopulation of tumor cells, namely cancer stem cells (CSCs) [23, 31]. Previous studies have confirmed that functional markers of CSCs in HNSCC include CD44, CD133, BMI1, SOX2 and ALDH1A1, etc. [40–42]. It is worth mentioning that our study also showed that HOXB7 silencing significantly inhibited the expression of CD44, CD133, BMI1, SOX2 and ALDH1A1 in HNSCC cells. Moreover, the silence of HOXB7 reduced the tumor ball formation ability of HNSCC cells. It suggested that HOXB7 was involved in regulating the expression of stemness-related genes. However, detailed regulatory targets of HOXB7 during HNSCC initiation and progression are still needed. In Pharmacology, HOXB7 acts as an ER co-factor, regulating the role of numerous ER targets including HER2 in tamoxifen-resistant breast cancer [43]. With HNSCC researches going deep, PD1 blockade therapy or combined with other chemotherapy has significantly changed the therapeutic landscape of HNSCC [44]. So, in this study, the CMap analysis was performed to predicate three small molecule drugs that could potentially be targeted as a therapeutic drug HOXB7, further suggesting that HOXB7 could be a novel therapeutic target in HNSCC. Of note, our data from genetic depletion of HOXB7 and other reports from genetic inhibition of HOXB7 support the notion that HOXB7 might be a novel and viable target, which can be therapeutically manipulated to treat cancer. In summary, combined with cell assay in vitro and vivo extremely support the idea that HOXB7 is a newly oncogene and therapeutic target in HNSCC. Our newly coverage together with previous findings above point out that not only HOXB7 serves as a novel diagnostic and prognostic cancer biomarker, but also shows tremendous potential as a therapeutic target.

## Conclusions

Our findings revealed the expression pattern, prognostic and tumorigenic roles of HOXB7 and identified HOXB7 as a novel biomarker with diagnostic and prognostic significance in HNSCC. However, limited number of patients examined and retrospective nature of our study precluded the unequivocal demonstration of prognostic and diagnostic significance of HOXB7 in HNSCC. A large amount of prospectively enrolled patients are required to convincingly establish its prognostic and diagnostic utility in HNSCC. Moreover, there is strong evidence already that HOXB7 plays a dominant role in facilitating tumor progression in HNSCC. If future work confirms its utility as a biomarker and its possible facile detection in easily accessible tissues (plasma, serum, sputum, and urine et al.), HOXB7 could eventually represent a fundamental tool for diagnosis and prognostic prediction of HNSCC. In addition, our findings also showed that HOXB7 as a putative oncogenic mediator underlying HNSCC initiation and progression, which suggested that selective targeting of HOXB7 by genetic or chemical approach might hold translational promise against but to further explore this, more work on finding out the exact HOXB7 regulatory network need be down in the future as well as novel inhibitors targeting HOXB7.

## Supplementary Information

**Additional file 1: Fig. S1.** **A** CMap analysis with the screening conditions under mean connective score < − 0.2 and *P* < 0.05, and finally screened 3 small molecule drugs potentially effective targeting HOXB7; B, D The chemical molecular structure of NU-1025 (Mean connective score=-0.560; *P* = 0.003) (**B**), thiamine (Mean connective score=-0.550; *P* = 0.038) (**C**), vinburnine (Mean connective score=-0.410; *P* = 0.018) (**D**) are shown.

**Additional file 2:  Fig. S2.** The workflow and analytical pipeline of the wholestudy.

## Data Availability

All data generated or analyzed during this study are included in this published article and its additional files. All original data are available upon request.
