# Supplementary figures and images for: HOXB7 acts as an oncogenic biomarker in head and neck squamous cell carcinoma
Source: Cancer Cell Int. 2021 Jul 24;21:393. doi: 10.1186/s12935-021-02093-6 (PMC8306226; doi:10.1186/s12935-021-02093-6)

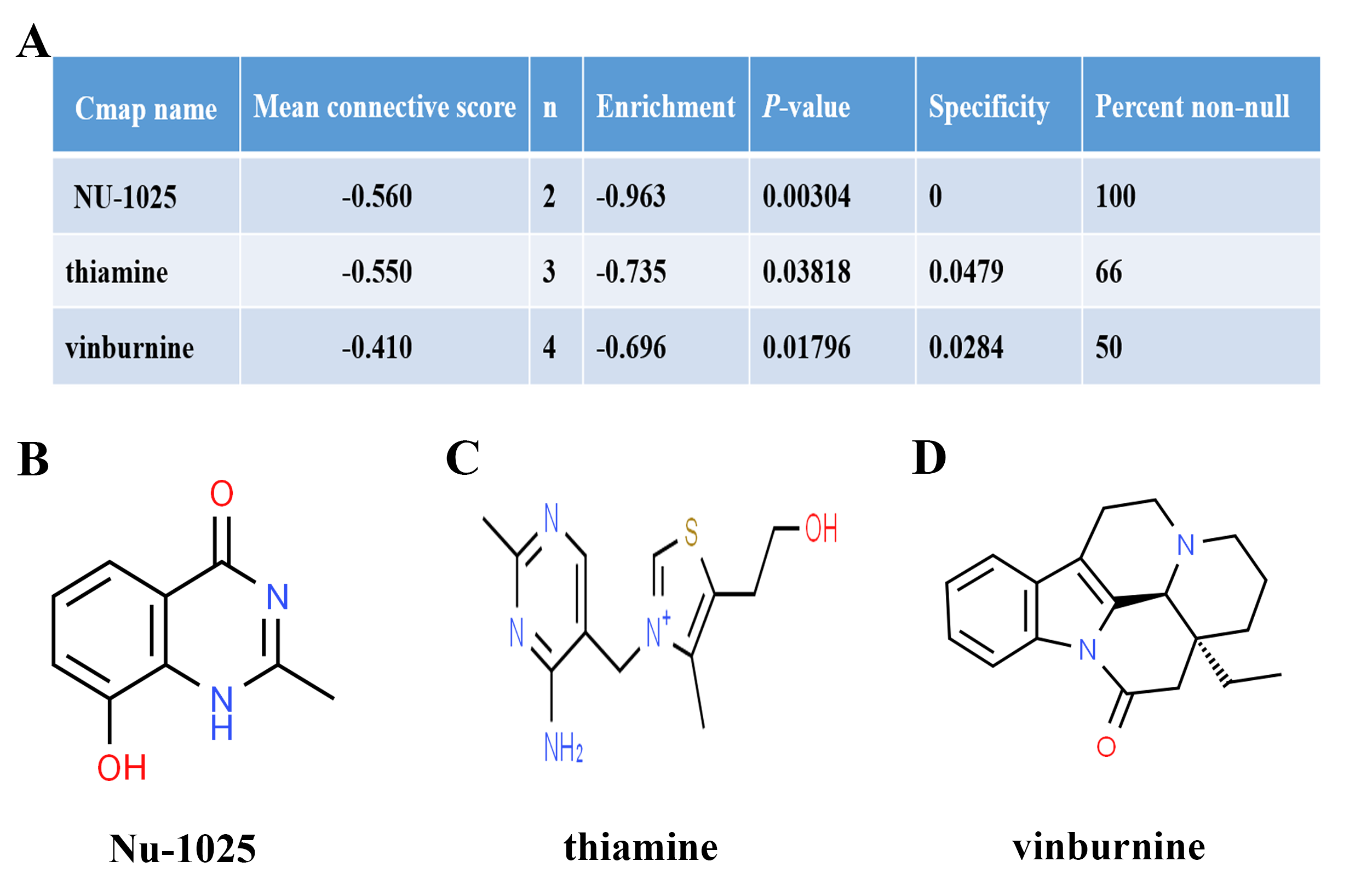

Supplement: Supplementary file 1 — Additional file 1: Fig. S1. A CMap analysis with the screening conditions under mean connective score < − 0.2 and P < 0.05, and finally screened 3 small molecule drugs potentially effective targeting HOXB7; B, D The chemical molecular structure of NU-1025 (Mean connective score=-0.560; P = 0.003) (B), thiamine (Mean connective score=-0.550; P = 0.038) (C), vinburnine (Mean connective score=-0.410; P = 0.018) (D) are shown. [file 12935_2021_2093_MOESM1_ESM.tif]

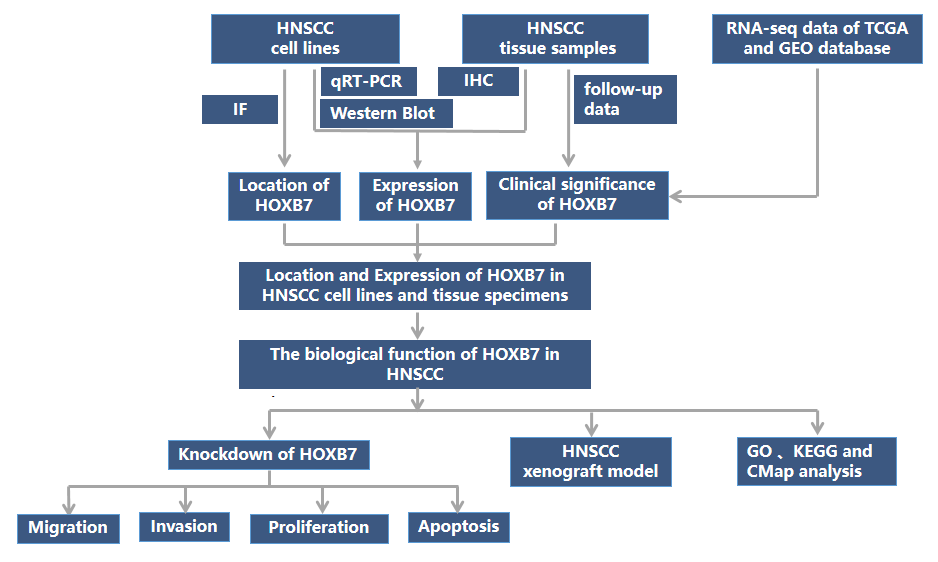

Supplement: Supplementary file 2 — Additional file 2: Fig. S2. The workflow and analytical pipeline of the wholestudy. [file 12935_2021_2093_MOESM2_ESM.tif]
